# An Herbal Formula Alleviates Lipotoxicity and Glucotoxicity by Regulating AMPK/SIRT1 in Zucker Diabetic Fatty Rats

**DOI:** 10.2174/0118715303402719251023002431

**Published:** 2026-01-20

**Authors:** Yajing Pan, Qiue Zhang, Yuhua Jiang, Xi Chen, Yuyan Huang, Jinsheng Yang

**Affiliations:** 1 Institute of Basic Theory for Chinese Medicine, China Academy of Chinese Medical Sciences, Beijing, 100700, China;; 2 School of Traditional Chinese Medicine, Beijing University of Chinese Medicine, Beijing, 102488, China

**Keywords:** Diabetic rats, Chinese herbal formula, AMPK, SIRT1, lipotoxicity, Qi transformation theory

## Abstract

**Introduction:**

This study aimed to investigate the effects of a Chinese herbal formula on the regulation of hepatic lipid metabolism in an inbred rat model of diabetes.

**Methods:**

ZDF *(fa/fa)* rats were administered the herbal formula for six weeks following a five-week feed of a high-fat diet. Blood glucose was monitored weekly. An oral glucose tolerance test (OGTT) and magnetic resonance imaging (MRI) were performed before sacrifice. The profiles of lipid and glucose in serum were determined, and the transcriptomic analysis was conducted. AMPK signaling was detected by PCR, western blot, and immunohistochemistry.

**Results:**

The herbal formula improved glucose tolerance and insulin sensitivity, reduced plasma glucose and lipid, alleviated pathological changes, and decreased hepatic lipid accumulation. Transcriptomic analysis predicted that AMPK signaling was a key pathway through which the herbal formula regulated hepatic lipid metabolism. The herbal formula upregulated the expressions of AMPKα, SIRT1, and PGC1α in the liver of ZDF rats.

**Discussion:**

The obese ZDF (*fa/fa*) rat, characterized by hyperglycemia, hyperlipemia, insulin resistance, and hepatic steatosis, closely mimics human T_2_DM. AMPK signaling plays a significant role in hepatic lipid metabolism, especially in the process of gluconeogenesis and glycogen synthesis. The herbal formula Qi Hua Tong Tiao is derived from ancient Chinese medical books in light of the Qi transformation theory. It alleviated lipotoxicity, glucotoxicity, and regulated hepatic lipid metabolism by up-regulating AMPK signaling. This study provided experimental evidence supporting the herbal formula as a promising alternative therapy for lipid disorders in diabetes.

**Conclusion:**

The Chinese herbal formula alleviated lipotoxicity and glucotoxicity by regulating AMPK/SIRT1 in the liver of ZDF rats.

## 1 INTRODUCTION

Diabetes mellitus (DM) is one of the fastest-growing global health emergencies, with an estimated worldwide population of 537 million in 2021 [1, 2]. It is reported that about 1 in 10 adults worldwide has DM. Type 2 diabetes (T_2_DM), the most common type of diabetes, accounts for up to 90% of the total. T_2_DM interferes with the ability to process and absorb glucose and is associated with multiple comorbidities and complications, which pose a major global health threat and impair the quality of life [3]. T_2_DM is a leading risk factor for cardiovascular mortality and imposes a substantial socioeconomic burden [3].

T_2_DM is a chronic noncommunicable disease that occurs when the pancreas produces insufficient insulin or the body cannot effectively utilize insulin [4]. Perturbations in glucose and lipid metabolism, which are one of the vital metabolic disturbances that develop over the course of T_2_DM, have detrimental effects on vessels throughout the whole body, leading to organ dysfunction and premature death [1]. The liver plays a core role. Abnormal hepatic glucose and lipid metabolism, and hepatic insulin resistance are central pathologic hallmarks of T_2_DM [5, 6]. When there is abnormal hepatic glycogen decomposition, gluconeogenesis, and hepatic glucose consumption, the dynamic balance between them is disrupted, resulting in hyperglycemia, insulin resistance, and diabetes [7]. Insulin resistance promotes hepatic glucose production and decreases glucose uptake, thus creating a vicious cycle [8].Therapy for hepatic dyslipidemia, steatosis, and insulin resistance is crucial for improving whole-body glucose and lipid homeostasis in diabetes [9, 10].

Currently, there is no cure for T_2_DM [11]. The main target in diabetes care is to maintain blood glucose levels in a healthy range and avoid long-term diabetes complications [4]. Appropriate drug therapy is crucial for delaying the development of T_2_DM and mitigating the complications and mortality burden [1, 3]. Evidence-based studies have proven that traditional Chinese herbs are effective and alternative therapies for T_2_DM [12-15]. Chinese herbs are reported to be effective in improving insulin sensitivity, alleviating the destruction of islet β cells, relieving symptoms with a few side effects, and improving quality of life [15-17]. The molecular mechanisms are closely associated with antioxidation, apoptosis regulation, hypoxia improvement, autophagy, and promotion of glucose and lipid metabolism [18]. In light of the Qi transformation theory in the oldest surviving Chinese medical classic, a clinically effective Chinese herbal formula, Qi Hua Tong Tiao, was administered to the Zucker diabetic fatty (ZDF) rats in this study. The profiles of hepatic lipid and glucose were determined to investigate the effects of the Chinese herbal formula in the spontaneous DM rat models.

## 2 MATERIALS AND METHODS

### 2.1 Reagents

The herbal formula Qi Hua Tong Tiao is composed of eight herbs (as detailed in Table **1**). The herbal formula was prepared by gently boiling the aforementioned crude drugs in the ratio of 8:3:8:4:4:2:8:2 in water for 60 minutes, then concentrating the solution using an R-220 Pro rotavapor (Buchi, Switzerland) and storing it at -80°C. The solution will be diluted to a density of 1.053 g/mL when used. All herbs were supplied by Yanjing Sliced Medicinal Herbs Factory (Beijing, China).

### 2.2 Study Design

16 seven-week-old specific pathogen-free male obese ZDF (*fa/fa*) rats with body weights of 280 ±1 4 g, and 8 age- matched heterozygous lean ZDF (*fa/+*) rats with body weights of 380 ±2 1 g were obtained from Vital River Laboratory Animal Technology Co., Ltd (Beijing, China) under animal license number SCXK (Jing) 2021-0017. Rats were housed in a temperature-controlled room at 23 ± 3°C and 40-70% relative humidity with a 12 h-12 h light-dark cycle in the laboratory animal center.

All rats were provided with ad libitum access to water and food. ZDF (*fa/fa*) rats were fed with a high-fat diet (Keaoxieli, Beijing, China) with 23.5% crude protein and 6.5% crude fat for five weeks to develop diabetes. After the five weeks of the designated diet, fasting blood glucose (FBG) of the lateral tail vein was measured using an Accu-Chek portable glucometer (Roche, Basel, Switzerland). Rats with FBG≥11.1 mmol/L were defined as diabetic rats. The ZDF (*fa/fa*) rats were assigned randomly into the diabetes mellitus group (DM, n=8) and the herbal formula group (H, n=8) according to the levels of blood glucose. Eight ZDF (*fa/+*) were used as the control ones (CON, n=8). The sample sizes were calculated using the G*power software (version 3.1.9.7, Franz Faul, Kiel University, Germany) based on a prior study and approved by the laboratory animal center of the Institute of Basic Theory for Chinese Medicine Science, China Academy of Chinese Medicine Science, in compliance with ARRIVE guidelines. The herbal formula at a dosage of 10.53 g/kg was gavaged daily to the rats of the H group in the next six weeks, while distilled water was gavaged to rats of the CON and the DM group. The dosage of the herbal medicine in this study was determined based on a pre-experiment (Table **S1**).

### 2.3 Blood Glucose Levels, Body Weight, Food and Water Intake Monitoring

FBG was monitored during the six weeks of administration. Briefly, blood samples were collected from the lateral tail vein of rats after the rats’ overnight fast at the same time every week. Blood glucose was measured using the portable glucometer mentioned above (as detailed in section 2.2). Body weight, water, and food intake were also monitored at the same time every week using an electronic balance (Yingheng, Guangdong, China).

### 2.4 Magnetic Resonance Imaging (MRI)

Three days before the sacrifice, three random rats per group were subjected to MRI by a Varian 7 Tesla/16 cm horizontal bore micro-MRI System (Agilent Technologies, CA, USA). Rats were placed in a supine position on the operating table by an experienced technician. Abdomen imaging was performed by fast spin-echo imaging sequence with a field of view (FOV) of 100*100 mm; 67 slices with 1.2-mm thickness; matrix size of 256*256; repetition time = 1298.46 ms; echo time = 13.21 ms; and 6 averages. The distribution of abdominal fat was observed. The volume of abdominal fat was quantified by marking ROIs on a per-voxel basis using Inveon Research Workplace 4.2 (Siemens Medical Solutions, PA, USA), and the ratio of abdominal fat volume to body weight was calculated [19].

### 2.5 Oral Glucose Tolerance Test (OGTT)

OGTT was performed two days before the sacrifice. After overnight fasting, the rats were gavaged with D-glucose solution (50%) at a dosage of 2 g/kg. Blood glucose was measured at regular intervals (0, 30, 60, 90, and 120 minutes) using the portable glucometer. Glucose tolerance was evaluated by calculation of the area under the curve (AUC) using the following equation (1):


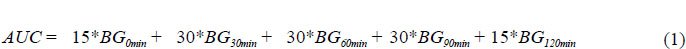


### 2.6 Biochemical Analyses

At the end of the 6^th^ week, the rats were sacrificed after an overnight fast. Blood was collected *via* abdominal aorta, and serum was separated by centrifugation at 3000 rpm for 10 minutes. Serum insulin, adiponectin, and leptin concentrations were determined by ELISA kits (E-EL-R2466c, E-EL-R3012, E-EL-R0582c; Elabscience, Wuhan, China) as per the manufacturer’s instructions separately. Serum nonesterified fatty acids (NE7001, Leadman, Beijing, China), triglyceride, cholesterol, ALT, AST, urea nitrogen, and creatinine (OSR61118, OSR6116, OSR6107, OSR6109, OSR6134, OSR6178; Beckman, Brea, CA, USA) concentrations were determined by commercial kits with an AU480 chemistry analyzer (Beckman Coulter, CA, USA). Insulin sensitivity was evaluated by calculation of the homeostasis model assessment of insulin resistance (HOMA-IR). HOMA-IR was calculated with FBG and fasting serum insulin (FINS) using the following equation (2):


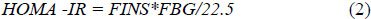


### 2.7 Morphological Observation

The liver and pancreas were harvested before being fixed with 4% formaldehyde for 48 hours, embedded in paraffin. Subsequently, sections of the liver and pancreas were deparaffinized and rehydrated before being stained with H&E. For the liver, parts of the paraffin sections were stained with periodic acid-schiff (PAS) to show glycogen accumulation, and parts of the sections from frozen tissue were stained with oil red O (ORO) to show neutral lipids and lipid droplet deposits. Morphological changes of the pancreas and liver were observed using an Aperio VERSA 8 System (Leica Biosystems, CA, USA) at 20× magnification.

### 2.8 Transcriptomic Analysis

#### 2.8.1 RNA Extraction and Library Preparation

Total RNA was prepared and extracted with TRIzol reagent, and then identified and quantified with a Qubit fluorescence quantifier and a Qsep400 high-throughput bio-fragment analyzer, respectively. mRNA was enriched and then cleaved into fragments. Specific cDNA was synthesized, followed by end repair, dA-tailing, and Sequencing adapter ligation, subsequently. After purification and fragment selection, DNA was amplified by PCR. After cDNA library preparation, the liver transcriptome was sequenced with 150 bp pair-ends reagents using Illumina HiSeq^TM^ 2500 by Metware Biotechnology Inc. (Wuhan, China).

#### 2.8.2 Data Analysis

Reading Quality was controlled using fastp to remove the adaptor sequences. The clean reads with high quality were aligned by HISAT to the reference genome. Levels of each gene were quantified. The FPKM reads of the gene were calculated, and the read counts were obtained. A principal component analysis was performed after data normalization with DESeq2. Expressions of genes with a significance level of *P* < 0.05 and a log_2_ (fold-change)≥ 1 were considered as differentially expressed genes (DEGs). Hierarchical cluster analysis, KEGG pathway enrichment of DEGs was performed with R software (edition 3.5.1).

### 2.9 Polymerase Chain Reaction (PCR)

TRIzol reagent was used for the extraction of total RNA from the liver. cDNA was prepared with a HiScript III RT SuperMix reverse transcriptase kit (Vazyme, Nanjing, China) by a T100 thermal cycler system (Bio-Rad, CA, USA). Quantitative reverse transcription PCR was conducted with a Taq Pro universal SYBR qPCR master mix (Q712, Vazyme, Nanjing, China) by a real-time PCR system (Applied Biosystems, MA, USA). Rat-specific PCR primers were designed according to the NCBI sequence database. The sequences were as follows: *Ampkα*, forward 5’-TCT GCC GTG GAC TAC TGT CA-3’, reverse 5’-AGA GAG TCC GAA GTC AGC TA-3’; *Sirt1*, forward 5’-ACT TGC CAT CAT GAA GC-3’, reverse 5’-TTA CTT TCA GAG AAG ACC CAA-3’; *Pgc1α*, forward 5’-TTC ATT ACC TAC CGT TAC ACC T-3’, reverse 5’-AGT TTC ATT CGA CCT GCG TA-3’; *Gapdh*, forward 5’-AAG ATG GTG AAG GTC GGT GT-3’, reverse 5’-GCT TCC CAT TCT CAG CCT TG-3’. The PCR results were expressed as the relative expression ratio of targeted genes and *Gapdh*.

### 2.10 Western Blot

The shredded liver was lysed in a lysis buffer supplemented with phosphatase and protease inhibitor cocktails (HY-K0021, HY-K0022, HY-K0010; MedChemExpress, NJ, USA), and ground to a homogenate. The concentration of protein in the supernatant was determined using a BCA protein assay kit. Subsequently, total proteins were separated by SDS-PAGE gel electrophoresis and then transferred to PVDF membranes. The membranes were blocked with blocking solution for over 1 hour at room temperature, followed by incubation with primary antibodies against AMPK (1:1000; 5831, CST, MA, USA), SIRT (1:1000; ab189494, Abcam, MA, USA), PGC1α (1:1000) or β-actin (1:1000; 4970, CST, MA, USA) at 4°C overnight. The species-specific secondary antibodies were used for subsequent incubation on the next day. Immunoblots were visualized with an ECL solution. The images were acquired using a Universal Hood Imaging System (Bio-Rad, CA, USA), and the blots were analyzed using ImageJ software (NIH, MD, USA).

### 2.11 Immunohistochemistry (IHC)

Sections of the liver were deparaffinized and rehydrated as mentioned above (as detailed in section 2.7). The sections of the liver were boiled in sodium citrate buffer (10 mM) for 12 min for antigen retrieval and incubated in goat serum for 30 min at 37°C for blocking, followed by incubation with primary antibodies against AMPKα (1:200; 66536-1-Ig, Proteintech, Wuhan, China), Sirt1 (1:300; ab189494, Abcam, MA, USA) and PGC1 (1:200; 66369-1-Ig, Proteintech, Wuhan, China) at 4°C overnight, separately. The species-specific secondary antibodies were used for subsequent incubation, and DAB was used for immunoreactivity visualization the next day. An Aperio VERSA 8 System (Leica Biosystems, CA, USA) was used to observe the liver sections and to acquire images at 40× magnification. Image J software (NIH, MD, USA) was used to analyze the sections. The average optical density (AOD) was calculated with integral optical density (IOD) using the following equation (3):


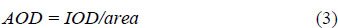


### 2.12 Statistical Methods

SPSS software (edition 20.0; IBM, NY, USA) was used for analyzing data. Continuous data were presented as mean ± standard error of the mean. Normality test was performed by the Shapiro-Wilk test. The significance of data with normal distribution was determined by a one-way ANOVA test, while other types of data were determined by a non-parametric test. The difference was considered significant when *P* < 0.05.

## 3 RESULTS

### 3.1 The Chinese Herbal Formula QiHuaTongTiao Improved Glucose Tolerance and Insulin Sensitivity, and Lowered Plasma Glucose and Lipid in ZDF Rats

**Fig. (1):**
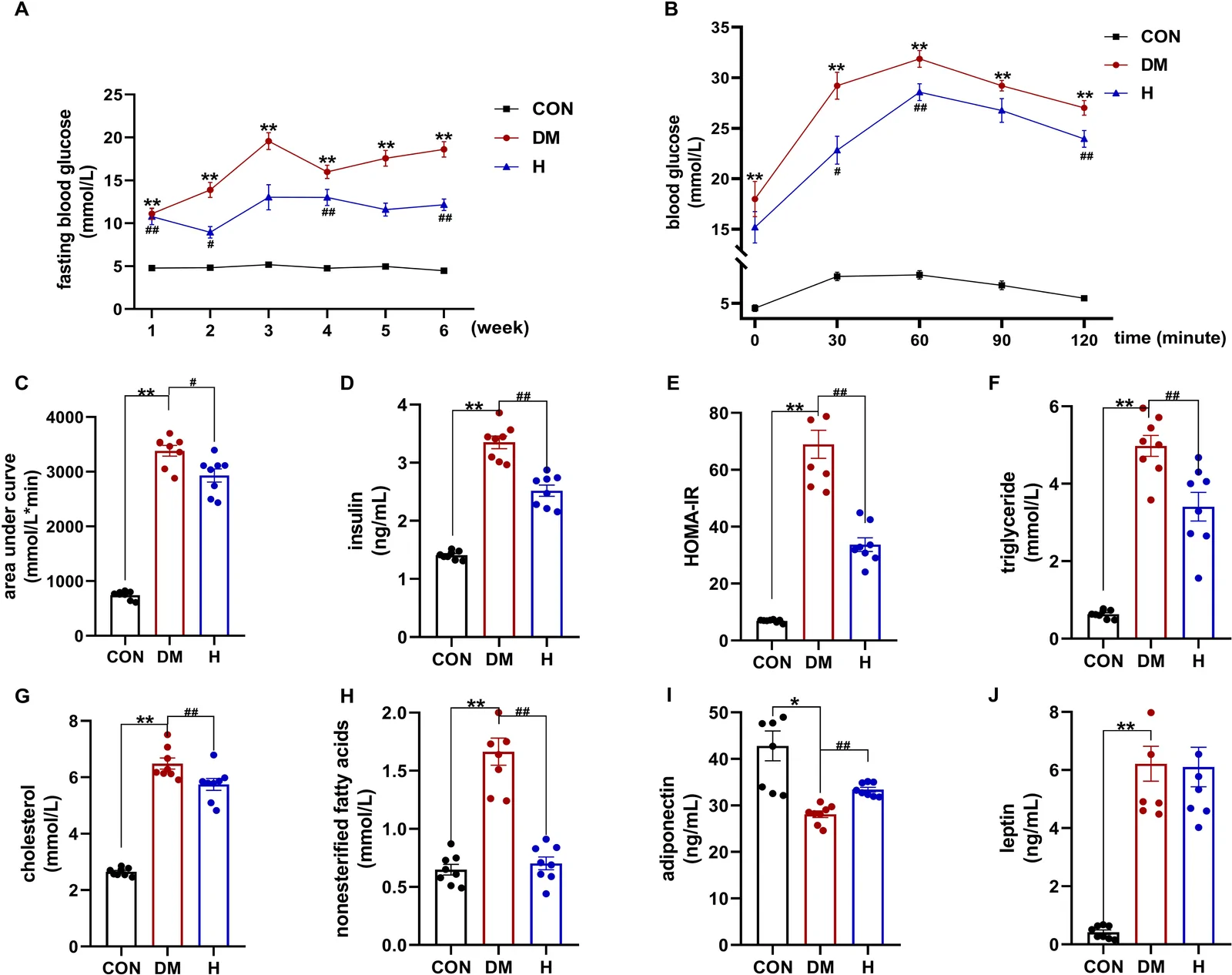
Laboratory assessments of ZDF rats. (**A**) FBG monitoring during the six weeks of herbal medicine administration. (**B**) Curves of OGTT. (**C**) AUC of OGTT. (**D**) Serum levels of insulin. (**E**) HOMA-IR. (**F-J**) Serum levels of triglyceride, cholesterol, nonesterified fatty acids, leptin, and adiponectin. Results are expressed as mean ± standard error, n=8, one-way ANOVA test. **P < 0.01 *vs.* the CON group; *P < 0.05 *vs.* the CON group; ^##^*P* < 0.01 *vs.* the DM group; ^#^*P* < 0.05 *vs.* the DM group.

FBG was monitored weekly, and OGTT was performed in the 6^th^ week. As shown in Fig. (**1A**), the average levels of FBG of rats in the DM group were significantly higher than those in the CON group (*P* < 0.01), and the average levels of FBG in the H group were significantly lower than those in the DM group (*P* < 0.01 or *P* < 0.05). The OGTT curves showed that the rats in the DM group had higher levels of glucose during the time-points of OGTT (Fig. **1B**) and had larger AUCs (Fig. **1C**) compared to the rats in the H group (*P* < 0.01). The rats in the H group had lower levels of glucose and had smaller AUCs compared to the rats in the DM group (*P* < 0.01 or *P* < 0.05). The serum levels of insulin (Fig. **1D**) and HOMA-IR (Fig. **1E**) of rats in the DM group were significantly higher than those in the CON group (*P* < 0.01). Additionally, the serum levels of insulin and HOMA-IR of rats in the H group were significantly lower than those in the DM group (*P* < 0.01). Collectively, these results suggested that the Chinese herbal formula improved glucose tolerance and insulin sensitivity in ZDF rats.

Serum levels of triglyceride (Fig. **1F**), cholesterol (Fig. **1G**), nonesterified fatty acids (Fig. **1H**), and leptin (Fig. **1I**) of rats in the DM group were significantly higher than those in the CON group (*P* < 0.01). Serum levels of triglyceride, cholesterol, and nonesterified fatty acids in the H group were significantly lower than those in the DM group (*P* < 0.01). Moreover, the serum level of adiponectin (Fig. **1J**) showed the opposite alteration among groups (*P* < 0.01 or *P* < 0.05). 

### 3.2 The Herbal Formula Decreased the Average Volume of Abdominal Fat in ZDF Rats Although the Difference was not Statistically Significant

**Fig. (2):**
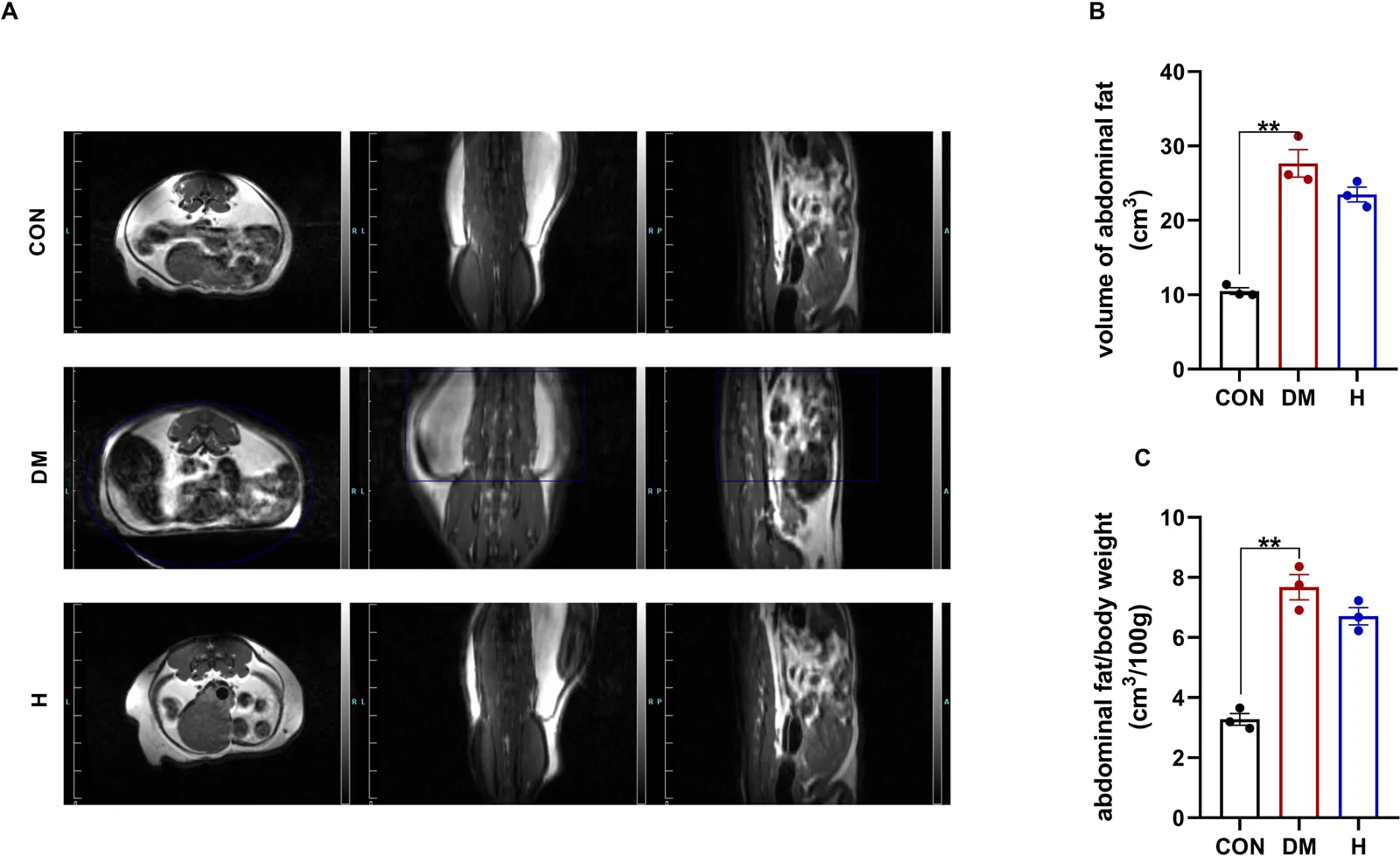
Abdominal fat of ZDF rats. **(A)** MRI of abdominal fat in the transverse, coronal, and sagittal planes. **(B)** Volume of the abdominal fat. **(C)** Relative volume of the abdominal fat (the ratio of abdominal fat volume to body weight). Results are expressed as mean ± standard error, n=3, one-way ANOVA test. **P < 0.01 *vs.* the CON group.

The distribution of abdominal fat was observed by MRI (Fig. **2A**). The average volume (Fig. **2B**) and the relative volume of abdominal fat (Fig. **2C**) of rats in the DM group were both significantly larger than those in the CON group (*P* < 0.01). The average volume of abdominal fat and the relative volume of abdominal fat in the H group were both smaller than those in the DM group, closely approaching statistical significance (23.71 ± 2.09 cm^3^ *vs.* 27.16 ± 3.64 cm^3^, *P* = 0.054; 6.78 ± 0.61 cm^3^/100g *vs.* 7.53 ± 0.75 cm^3^/100g, *P* = 0.074).

### 3.3 The Herbal Formula Alleviated Pathological Morphology of the Pancreas and the Liver, and Decreased Hepatic Lipid Accumulation in ZDF Rats

Fig. (**3A**) showed that the rats in the CON group had normal pancreatic tissue with lobular architecture and round, well-granulated islets. The rats in the DM group had degenerated and degranulated islets, while the rats in the H group had less islet degeneration and degranulation. The morphological changes of the liver among groups were observed using different staining methods. As shown in Fig. (**3B**), the rats in the CON group had a normal hepatic architecture with the portal tract surrounded by a radial pattern of hepatic lobules, and without distorted architecture or parenchymal collapse. There were numerous lipid droplets and vacuoles in the liver of rats in the DM group. The hepatic steatosis in rats of the H group was alleviated observably compared to that in the DM group. PAS staining highlights the glycogen content in the hepatocytes. As shown in Fig. (**3C**), compared to the rats in the CON group, there was much more accumulation of glycogen in the liver of rats in the DM group, with PAS staining dark purple-red. Furthermore, the rats in the H group had less accumulation of glycogen compared to those in the DM group, with PAS staining light purple. ORO is an accurate method for detecting lipid droplets in the hepatocytes and efficiently helps to visualize hepatic steatosis. As shown in Fig. (**3D**), rats in the DM group had extensive hepatic steatosis with ORO staining dark red. And the rats in the H group had less steatosis with ORO staining light orange-red. Collectively, the morphological changes suggested that the herbal formula partly alleviated pathological changes of the pancreas and the liver, and decreased hepatic lipid accumulation in ZDF rats to some extent.

### 3.4 The Herbal Formula Altered Transcriptomic Profiles in ZDF Rats. AMPK Signaling was Predicted to be one of the Critical Pathways in the Effects of the Herbal Formula on Regulating Hepatic Lipid Metabolism in ZDF Rats

**Fig. (4):**
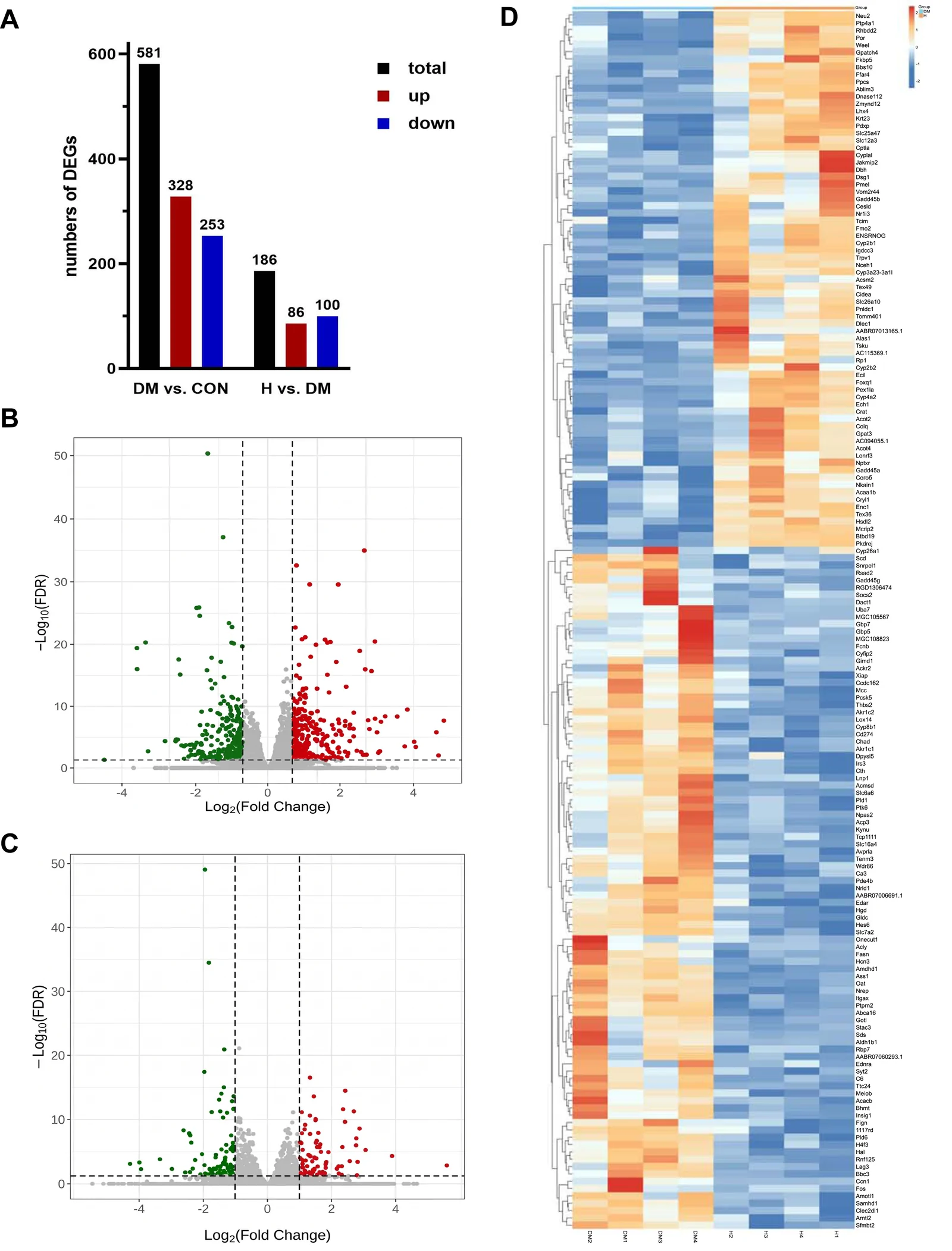
DEGs among groups. (**A**), bar chart of DEGs among groups (**B**), volcano plot of DEGs between the DM and the CON group (**C**), volcano plot of DEGs between the H and the DM group (**D**) n=4.

Transcriptomic analysis was performed to determine the effects of the herbal formula on hepatic transcription. A total of 708 DEGs were identified (Fig. **4A**), 328 were upregulated and 253 were downregulated in the DM group compared to the CON group (Fig. **4B**). Additionally, 86 were upregulated and 100 were downregulated in the H group compared to the DM group (Fig. **4C**). The clustering heatmap of the top DEGs between the H and the DM group were shown in Fig. (**4D**).

**Fig. (5):**
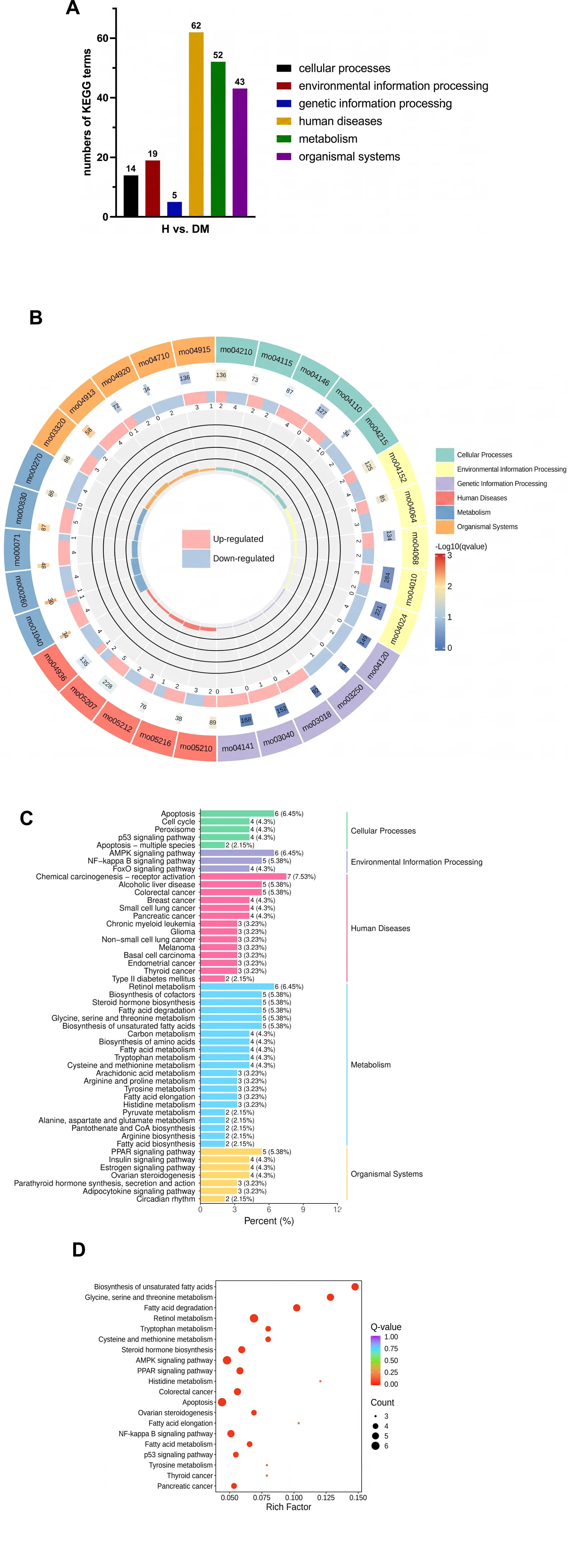
KEGG enrichment of DEGs between the H and the DM group. (**A**) Number of KEGG terms in different classifications. (**B**) Visualized circos plot of representative pathways with the lowest *q*-values in different classifications. (**C**) The fifty enriched pathways with the lowest *q*-values. (**D**) The twenty enriched pathways with the overall consideration of low *q*-values, high rich factors, and large counts of DEGs. n=4.

To further investigate how the herbal formula influences the hepatic transcription, KEGG pathway enrichment between the H and DM groups was analyzed. The results were shown in Fig. (**5**). In total, 195 pathways were enriched, 14 of which were classified as cellular processes, 19 as environmental information processing, 5 as genetic information processing, 62 as human diseases, 52 as metabolism and 43 as organismal systems, respectively (Fig. **5A**). The representative pathways were visualized as circos plot (Fig. **5B**). Fig. (**5C**) showed the fifty pathways with the highest enrichment and the lowest *q*-values, and Fig. (**5D**) showed the twenty pathways with the highest enrichment based on the overall consideration of *q*-values, rich factors and counts of DEGs. Among these typical pathways, AMPK signaling, which is closely involved in fatty acid metabolism, might play a critical role in the effects of the herbal formula on regulating hepatic lipid metabolism in ZDF rats.

### 3.5 The Herbal Formula Upregulated Expressions of AMPKα, SIRT1, and PGC1α of the Liver in ZDF Rats

To investigate how the herbal formula regulates hepatic AMPK signaling, PCR of the target genes, WB, and IHC of the target proteins were conducted subsequently. As shown in Fig. (**6A**), relative gene expressions of *Ampkα, Sirt1,* and *Pgc1α* in the H group were significantly higher than those in the DM group (*P* < 0.01). Fig. (**6B**) showed the protein bands, Figs. (**6C** and **6D**) showed the extent of dyeing and AOD of positive areas. The expressions of AMPKα, SIRT1, and PGC1α in the H group were significantly higher than those in the DM group (*P* < 0.01 or *P* < 0.05). Collectively, these results suggested that the herbal formula upregulated expressions of AMPKα, SIRT1, and PGC1α.

### 3.6 The Herbal Formula did not Significantly Change the Body Weight, Food, or Water Intake

**Fig. (7):**
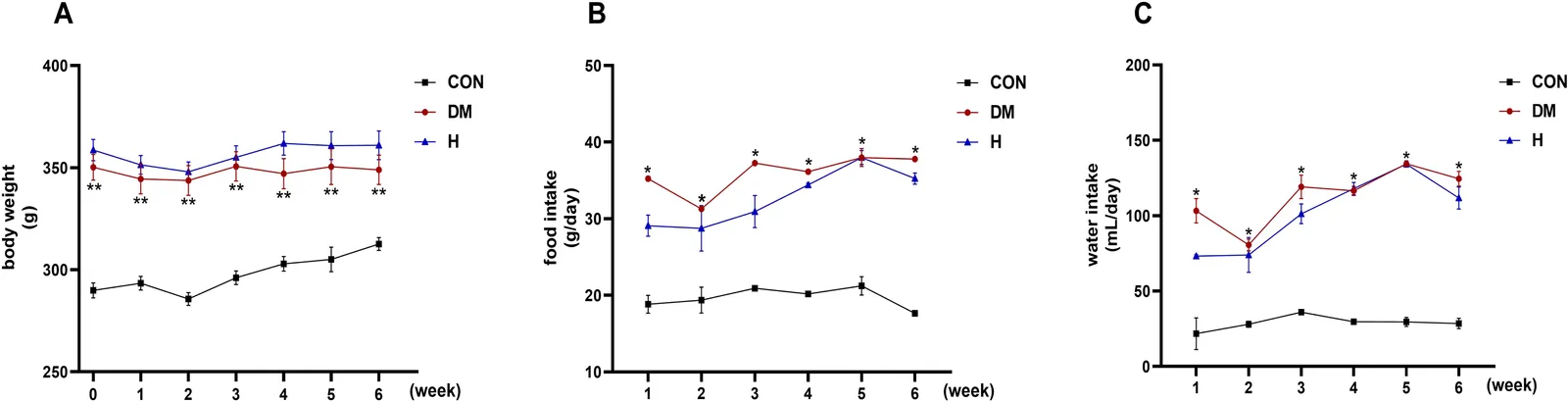
Body weight (**A**), food (**B**), and water intake (**C**) in ZDF rats. Results are expressed as mean ± standard error, n=8, one-way ANOVA test. **P < 0.01 *vs.* the CON group; *P < 0.05 *vs.* the CON group.

The average body weight of rats in the DM group was significantly greater than that in the CON group during the administration period (Fig. **7A**; *P* < 0.01). The average daily consumption of food (Fig. **7B**) and water (Fig. **7C**) in the DM group was significantly higher than that in the CON group (*P* < 0.05). There was no statistical significance in body weight, food, and water intake between the H and the DM group (*P* > 0.05).

### 3.7 The Herbal Formula did not Impair the Functions of the Liver and the Kidneys

**Fig. (8):**
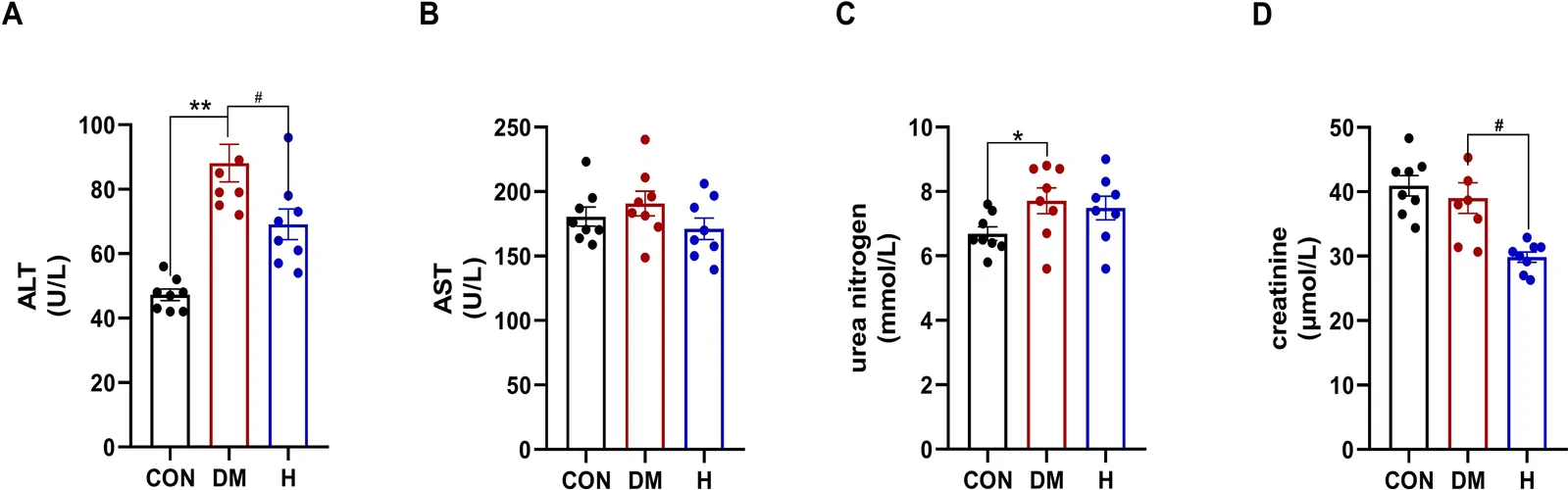
Serum levels of ALT **(A)**, AST **(B)**, urea nitrogen **(C)**, and creatinine **(D)** in ZDF rats. Results are expressed as mean ± standard error, n=8, one-way ANOVA test. ^**^P < 0.01 *vs.* the CON group; ^*^P < 0.05 *vs. *the CON group; ^#^*P* < 0.05 *vs.* the DM group.

AST and ALT were used to assess liver function. Urea nitrogen and creatinine were used to assess renal function. There were no significant increases in ALT (Fig. **8A**), AST (Fig. **8B**), urea nitrogen (Fig. **8C**), and creatinine (Fig. **8D**) of rats in the H group compared to those in the DM group. The herbal formula did not impair the functions of the liver and the kidneys in ZDF rats.

## 4 DISCUSSION

Lipid disorder (dyslipidemia) is a complicated and vital pathological change of T_2_DM. It is considered the upstream disease of T_2_DM [11]. Hepatic steatosis, characterized by the accumulation of lipids in hepatocytes, is usually associated with T_2_DM [20]. Diabetic patients frequently exhibit elevated levels of cholesterol, triglycerides, and LDL-cholesterol particles, a condition clinically referred to as diabetic dyslipidemia [10, 20]. When hepatic insulin resistance states occur in the process of diabetes, there are lipid disorders such as increased VLDL secretion, reduced VLDL clearance, reduced nonesterified fatty acid absorption, and enhanced lipolysis. These disorders themselves cause insulin resistance and islet β cell dysfunction, resulting in a vicious cycle of insulin resistance [21]. Therefore, it is essential to regulate lipid disorders in the process of diabetes.

In light of the Qi transformation theory in the oldest surviving Chinese medical classic, the *Yellow Emperor's Classic of Internal Medicine* [22, 23], the herbal formula QiHuaTongTiao was used in this study, which was modified from the Qihua Decoction reported in *Recording of Syndrome Differentiation* (an ancient medical book completed around 1687 A.D.) and the Zhizhu Pill are reported in *Revelation of Medicine* (an ancient medical book completed around 1186 A.D.). The herbal formula consists of eight herbs (Table **1**). *Astragalus mongholicus* is widely used in China because of the immunomodulatory, hypoglycemic, antioxidant, and anti-inflammatory effects [24-26]. More than 200 small- molecule chemical substances, containing polysaccharides, saponins, and flavonoids, have been identified [24]. The active compounds of *Astragalus mongholicus*, such as Astragalus polysaccharides, kaempferol, and quercetin, have been reported to reduce FBG levels, regulate β-cell proliferation and apoptosis, ameliorate insulin resistance and lipid accumulation, and modulate AMPK/SIRT1 and PI3K/AKT pathways in T_2_DM [24, 25]. Furthermore, the active compounds can attenuate mitochondrial damage and ameliorate vascular endothelial dysfunction in diabetic complications [24, 27]. The active compounds of *Atractylodes macrocephala*, such as volatile oils, lactones, and polysaccharides, have been reported to improve glycemia and lipid metabolism disorders, regulate insulin secretion, ameliorate insulin resistance, modulate immunity, modulate gut bacteria associated with metabolic syndrome, and inhibit inflammatory responses [28-30]. The active compounds of *Citrus aurantium *L. possess pharmacologic activities in regulating hepatic lipid metabolism and glycometabolism, inhibiting hepatocyte apoptosis and inflammation, and modulating AMPK/SIRT1 and MAPK/ NF-κB pathways [31, 32]. The active compounds of *Ligusticum chuanxiong *and *Angelica sinensis* also have anti-inflammatory, anti-diabetic, antioxidant, immune regulation, and hepatoprotective activities [33-36]. The herbal formula with abundant bioactive components might function in multi-target and multi-pathway modes. Further studies will characterize the bioactive components to elucidate the mechanistic basis for efficacy.

To determine the effects of the herbal formula on regulating glucose and lipid metabolism in diabetes, this study used male ZDF (*fa/fa*) rats with a high-fat diet. Male rather than female rats were employed in order to eliminate potential confounding effects caused by the estrous-cycle-driven estrogen level fluctuations. The obese ZDF (*fa/fa*) rat is an inbred rat model that carries a genetic mutation of the leptin receptor. It develops diabetes over time when fed with a high-fat diet, characterized by hyperinsulinemia, hyperglycemia, insulin resistance, hyperlipemia, and obesity [37, 38]. It closely mimics human T_2_DM and is widely used in studying the physiological events and the treatment of T_2_DM. In this study, the rats in the DM group had larger AUC in OGTT, higher serum levels of FBG, insulin, HOMA-IR, triglyceride, cholesterol, and nonesterified fatty acids compared to those in the CON group. This indicated that the obese ZDF (*fa/fa*) rats developed hyperglycemia, hyperlipemia, and insulin resistance, consistent with findings from other studies [39-41]. Compared to the rats in the DM group, the rats in the H group had lower serum profiles of glucose and lipid metabolism, suggesting that the herbal formula lowered serum lipid, improved glucose tolerance, and insulin sensitivity of ZDF rats.

ZDF rats developed hepatic steatosis, characterized by an excessive accumulation of lipid droplets inside the cytoplasm of hepatocytes [38]. The sections of the liver stained by different methods (H&E, PAS, and ORO staining) collectively showed that there was mild hepatic steatosis, with less accumulation of glycogen and lipid droplets in the rats in the H group, suggesting that the herbal formula alleviated pathological morphology and decreased hepatic lipid accumulation.

The amount of fat mass alters with the increase in lipid deposition. MRI is a rapid, visualized, and noninvasive tool for assessing fat distribution [42] and the gold standard for measuring visceral fat [43]. Therefore, the volumes of abdominal fat were calculated by MRI in this study. The results showed that the rats in the DM group had significantly higher volumes of abdominal fat than those in the CON group. However, the rats in the H group had smaller volumes of abdominal fat than those in the DM group, with a lack of statistical significance. This might partly result from a small sample size (randomly three rats per group), and we could not conclude that the herbal formula decreased the average volume of abdominal fat. A further study with a large sample size might be needed in future studies.

Further, to determine the mechanisms underlying the effects of the herbal formula QiHuaTongTiao, RNA was extracted from the liver, mRNA was enriched, and cDNA was synthesized and amplified. The DEGs were analyzed, and KEGG pathway enrichment was conducted. The results indicated that a total of 708 DEGs were identified, and 195 pathways were enriched. Among the most enriched and typical pathways, AMPK signaling received significant attention because of its vital role in the process of hepatic lipid metabolism. The liver controls the body’s glucose level through gluconeogenesis and glycogen synthesis. AMPK and SIRT are critical enzymes that sense intracellular energy status and regulate glucose and lipid metabolism. AMPK, the sensor of energy status, plays an important role in β cell secretion, glucose uptake, glycogen storage, hepatic fatty acid synthesis, and cholesterol synthesis [44-46]. The activation of AMPK decreases intracellular fat accumulation, attenuates hepatic steatosis, and regulates lipid and carbohydrate metabolism in liver cells [47]. SIRT1 regulates metabolism, including fat cell accumulation and maturation, and lipid metabolism in the liver [48-50].It has been proven that the overexpression of SIRT1 alleviated hepatic steatosis and glucose intolerance in an animal model of diabetes, while the silence of SIRT1 in the liver developed hepatic steatosis [48]. Moreover, SIRT1 and AMPK interact with each other and present a mutual reinforcement [51]. The co-activation of AMPK and SIRT1 contributes to reducing lipogenesis and alleviating hepatic steatosis. AMPK and SIRT1 are upstream modulators that activate PGC-1α and regulate mitochondrial homeostasis [52]. AMPK and SIRT1 are active and stabilize PGC1α, a transcription factor that promotes mitochondrial biogenesis and regulates nonesterified fatty acids oxidation, and thereby improve lipid metabolism in the process of insulin resistance and T_2_DM. Activated PGC1α subsequently interacts with downstream targets, initiating the transcription of a series of components that mobilize nonesterified fatty acids [53]. Therefore, AMPK has been proven to be a critical factor in glucose and lipid metabolism, and probably becoming a promising therapeutic target for diabetes [54-56]. Using the methods of PCR, western blot, and IHC, the expressions of AMPKα, SIRT1, and PGC1α of the liver in the H group were found to be significantly higher than those in the DM group, supporting that the herbal formula upregulated AMPK signaling.

Collectively, this study demonstrated that the herbal formula regulated hepatic glucose and lipid metabolism, carrying clinical implications for integrated diabetes management. It provides experimental evidence supporting its potential as an adjunctive therapy to conventional hypoglycemic agents, while establishing a foundation for future studies.

There were some limitations of this study. While this study focused on a single dosage of the herbal formula, subsequent work will quantify dose-dependent effects. Given the multi-component and multi-target nature of Chinese herbal formulas, subsequent work will characterize their bioactive ingredients to elucidate the mechanistic basis for their efficacy.

## CONCLUSION

This study demonstrated that the Chinese herbal formula Qi Hua Tong Tiao alleviated lipotoxicity, glucotoxicity, and regulated hepatic lipid metabolism in ZDF rats. The underlying mechanisms were partly associated with the upregulation of AMPK signaling. This study provided experimental evidence supporting the herbal formula as a promising alternative therapy for lipid disorders in diabetes.

## Figures and Tables

**Fig. (3):**
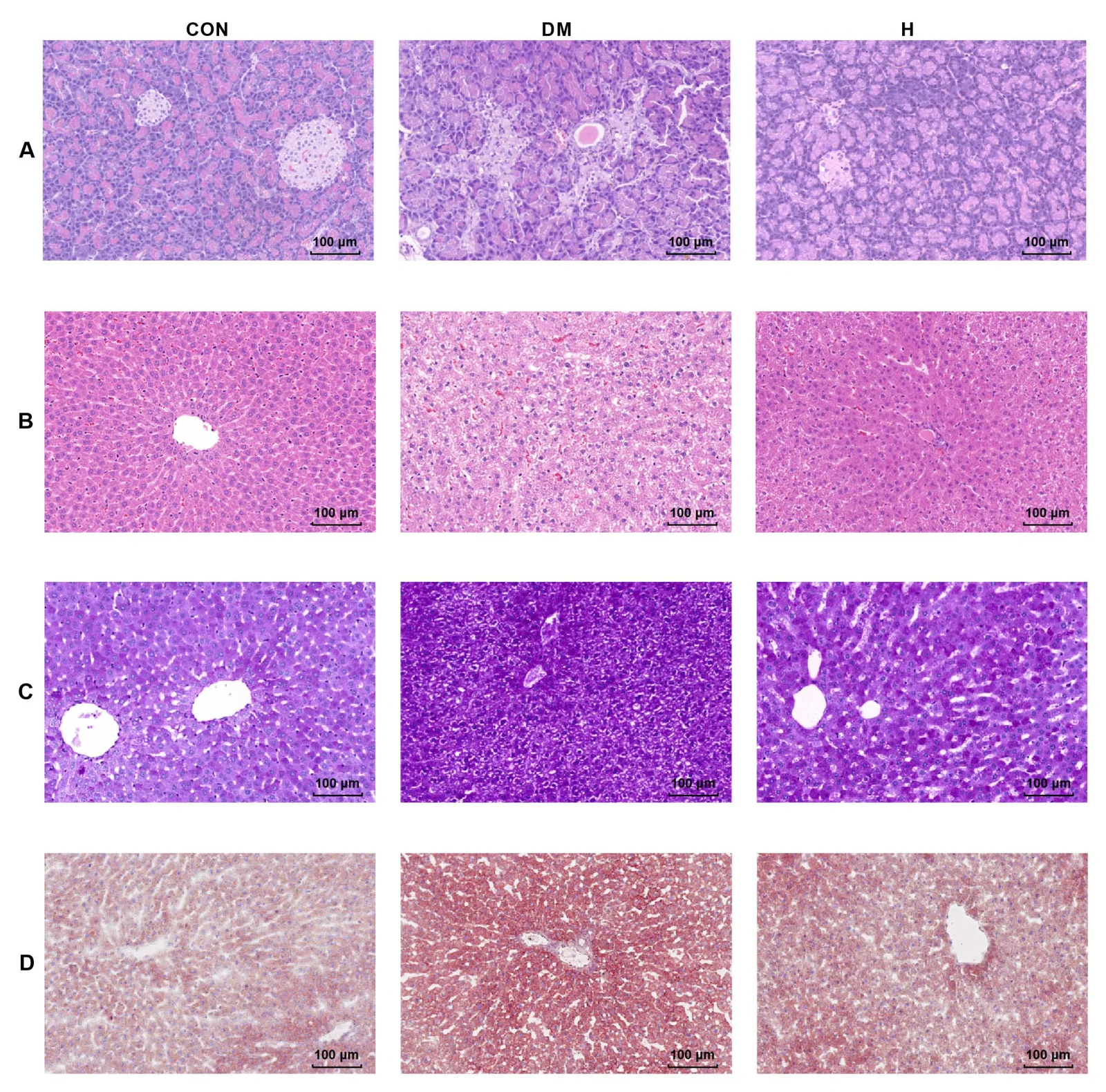
H&E staining of the pancreas sections **(A)** the liver sections **(B)**, PAS staining of the liver sections **(C)**, and ORO staining of the liver sections **(D)** in ZDF rats (×200).

**Fig. (6):**
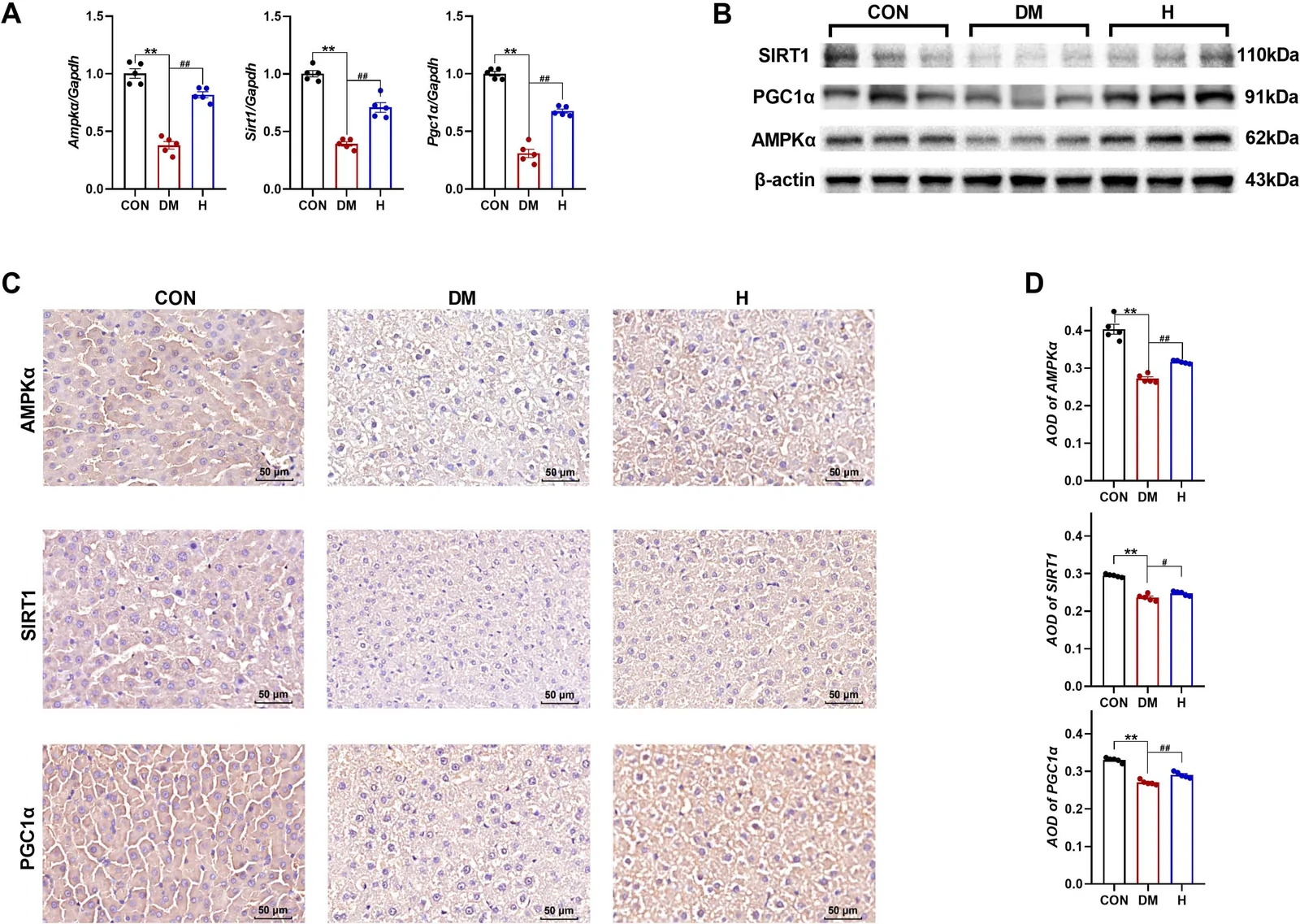
Expressions of AMPKα, SIRT1, and PGC1α of the liver. **(A)** Relative expression ratio of targeted genes and *Gapdh*. **(B)** Gray-scale images of targeted protein bands for Western blot. **(C** and **D)** Immunoreactive images and the AODs for immunohistochemistry (×400). Results are expressed as mean ± standard error, n=5 for PCR and IHC, n=3 for WB, one-way ANOVA test. **P < 0.01 *vs.* the CON group; ^##^*P* < 0.01 *vs.* the DM group; ^#^*P* < 0.05 *vs.* the DM group.

**Table 1:** The composition of the herbal formula Qi Hua Tong Tiao.

Latin Name	Pinyin	Family Name	used Part	LOT Number	Weight (g)
*Astragalus mongholicus* Bunge	Huangqi	Fabaceae	root	2308303	2.16
*Bupleurum falcatum* L.	Chaihu	Apiaceae	root	2310016	0.81
*Atractylodes macrocephala* Koidz.	Baizhu	Asteraceae	rhizome	2308030	2.16
*Polyporus*	Zhuling	-	sclerotium	2307006	1.08
*Angelica sinensis* (Oliv.) Diels	Danggui	Apiaceae	root	2309004	1.08
*Conioselinum anthriscoides 'Chuanxiong'*	Chuanxiong	Apiaceae	rhizome	2306028	0.54
*Citrus × aurantium* L.	Zhishi	Rutaceae	fruit	2307046	2.16
*Raphanus raphanistrum subsp. sativus* (L.) Domin	Laifuzi	Brassicaceae	seed	2305014	0.54

**Note:** The information was checked with https://mpns.science.kew.org/.

## Data Availability

The data and supportive information are available within the article.

## LIST OF ABBREVIATIONS

AMPKα: 5’-Adenosine Monophosphate-Activated Protein Kinase Alpha
AOD: Average Optical Density
AUC: Area Under Curve
DM: Diabetes Mellitus
FBG: Fasting Blood Glucose
FINS: Fasting Serum Insulin
HOMA-IR: Homeostasis Model Assessment of Insulin Resistance
IHC: Immunohistochemistry
IOD: Integral Optical Density
MRI: Magnetic Resonance Imaging
OGTT: Oral Glucose Tolerance Test
ORO: Oil Red O
PAS: Periodic Acid-Schiff
PGC1α: Peroxisome Proliferator-Activated Receptor Gamma Coactivator 1 Alpha
SIRT1: Sirtuin 1
T_2_DM: Type 2 Diabetes
ZDF: Zucker Diabetic Fatty
