# Supplementary material for: An Herbal Formula Alleviates Lipotoxicity and Glucotoxicity by Regulating AMPK/SIRT1 in Zucker Diabetic Fatty Rats
Source: Endocr Metab Immune Disord Drug Targets. 2026 Jan 20;26:E18715303402719. doi: 10.2174/0118715303402719251023002431 (PMC13527771; doi:10.2174/0118715303402719251023002431)

Supplementary Material

An Herbal Formula Alleviates Lipotoxicity and Glucotoxicity by Regulating AMPK/SIRT1 in Zucker Diabetic Fatty Rats

Yajing Pan[1,#, *], Qiue Zhang[2,#], Yuhua Jiang[1], Xi Chen[1], Yuyan Huang[1] and Jinsheng Yang[1,*]

[1] *Institute of Basic Theory for Chinese Medicine, China Academy of Chinese Medical Sciences, Beijing, 100700, China;*
[2]*School of Traditional Chinese Medicine, Beijing University of Chinese Medicine, Beijing, 102488, China*

Supplementary Table 1. Laboratory assessments of ZDF rats in the pre-experiment with three dosages of the herbal formula.

Group	FBG (mmol/L)	insulin (ng/mL)	AUC (mmol/L*min)	triglyceride (mmol/L)	Cholesterol (mmol/L)	Nonesterified Fatty Acids (mmol/L)
CON	4.250±0.263	1.458±0.028	694.875±40.214	0.640±0.056	2.563±0.050	0.678±0.066
DM	20.175±0.879**	3.540±0.106**	3558.750±50.729**	5.550±0.183**	6.660±0.302**	1.825±0.121**
H	12.200±0.867##	2.446±0.157#	3000.750±88.008##	3.068±0.599#	5.420±0.275##	0.698±0.107##
M	16.525±1.465#	2.723±0.207	3148.500±51.385#	3.203±0.492#	5.678±0.282#	1.183±0.104##
L	16.850±0.473#	2.452±0.067#	3235.125±187.763#	3.820±0.205	6.140±0.359	1.313±0.050##

Results are expressed as mean ± standard error, n=4, one-way ANOVA test for normally distributed data, and nonparametric test for non-normally distributed data. **$P < 0.01$ *vs.* the CON group; *$P < 0.05$ *vs.* the CON group; ##$P < 0.01$ *vs.* the DM group; #$P < 0.05$ *vs.* the DM group. H, herbal formula at a dosage of 10.53 g/kg; M, herbal formula at a dosage of 5.27 g/kg; L, herbal formula at a dosage of 2.63 g/kg.

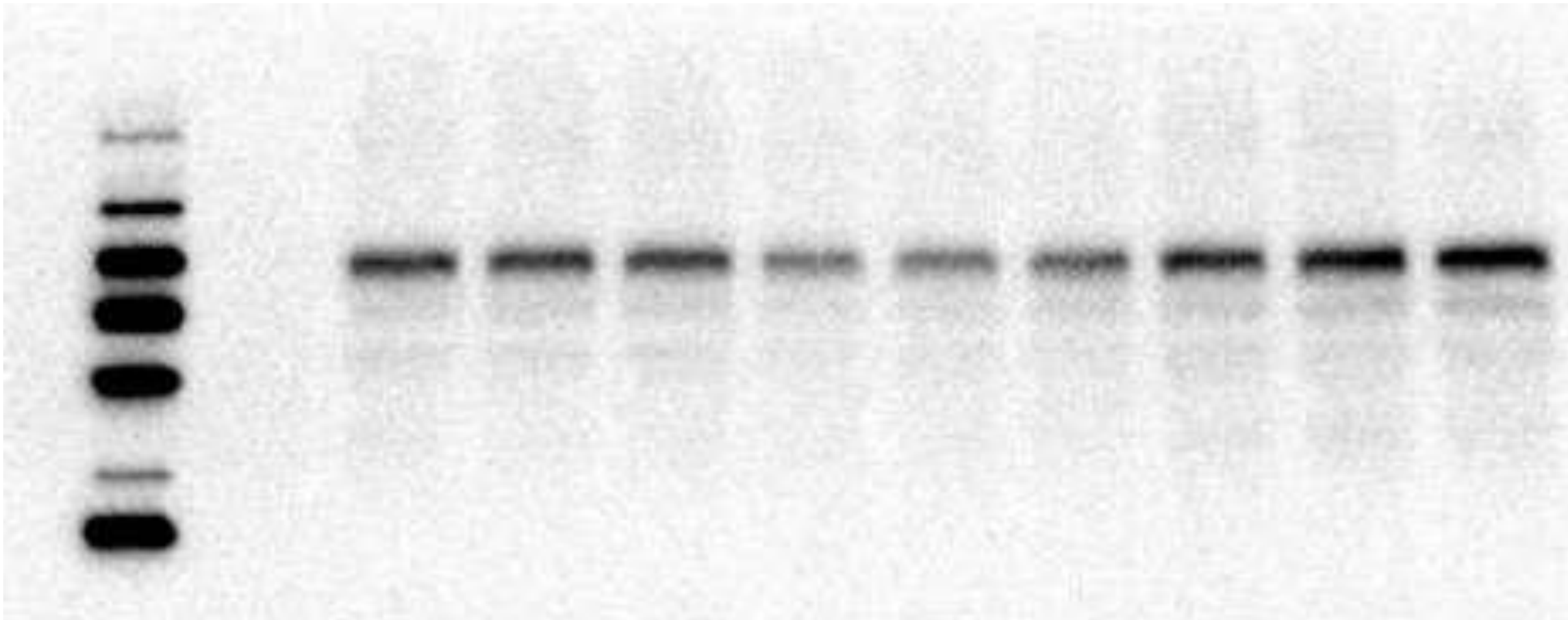

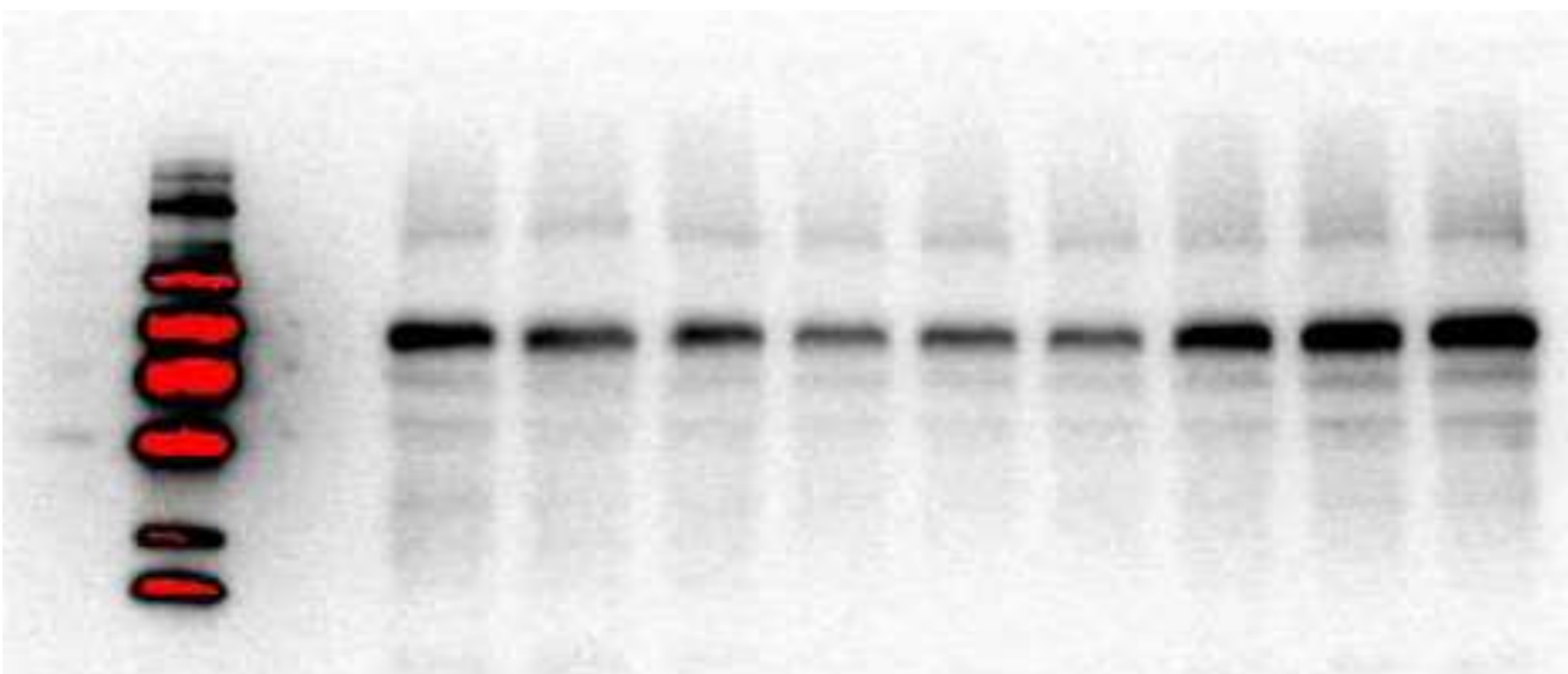

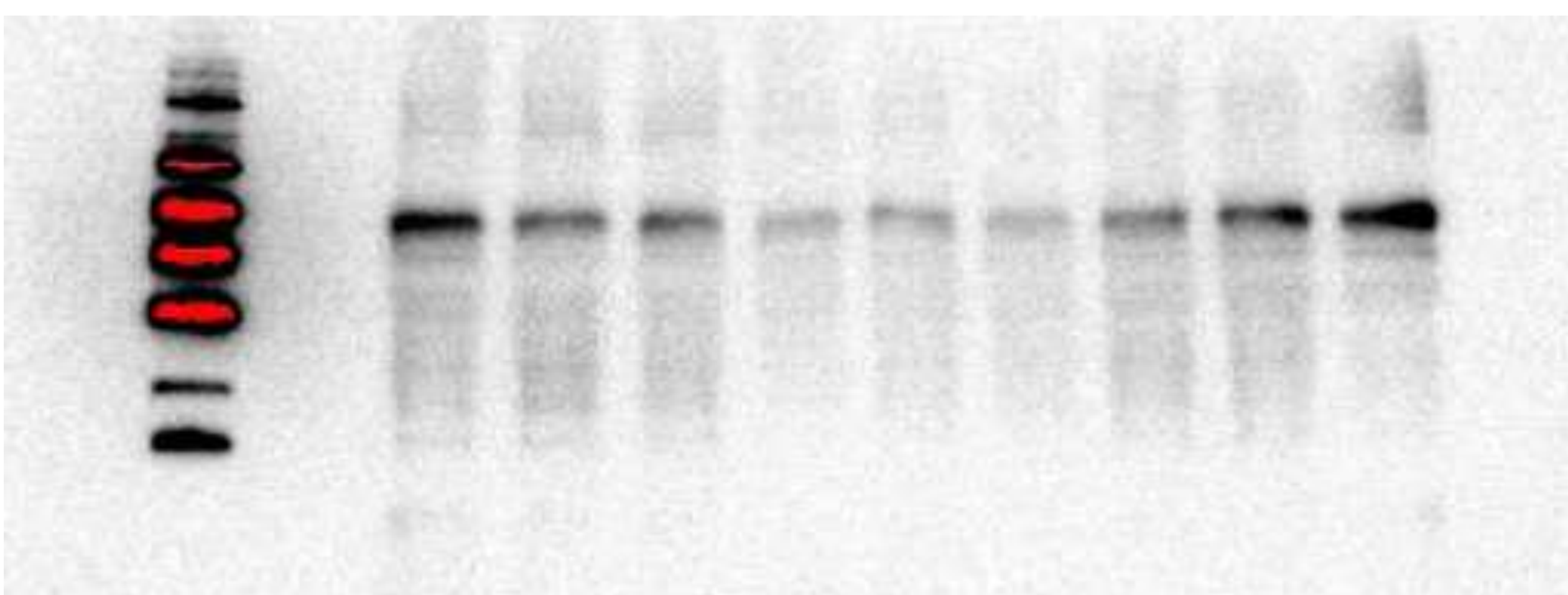

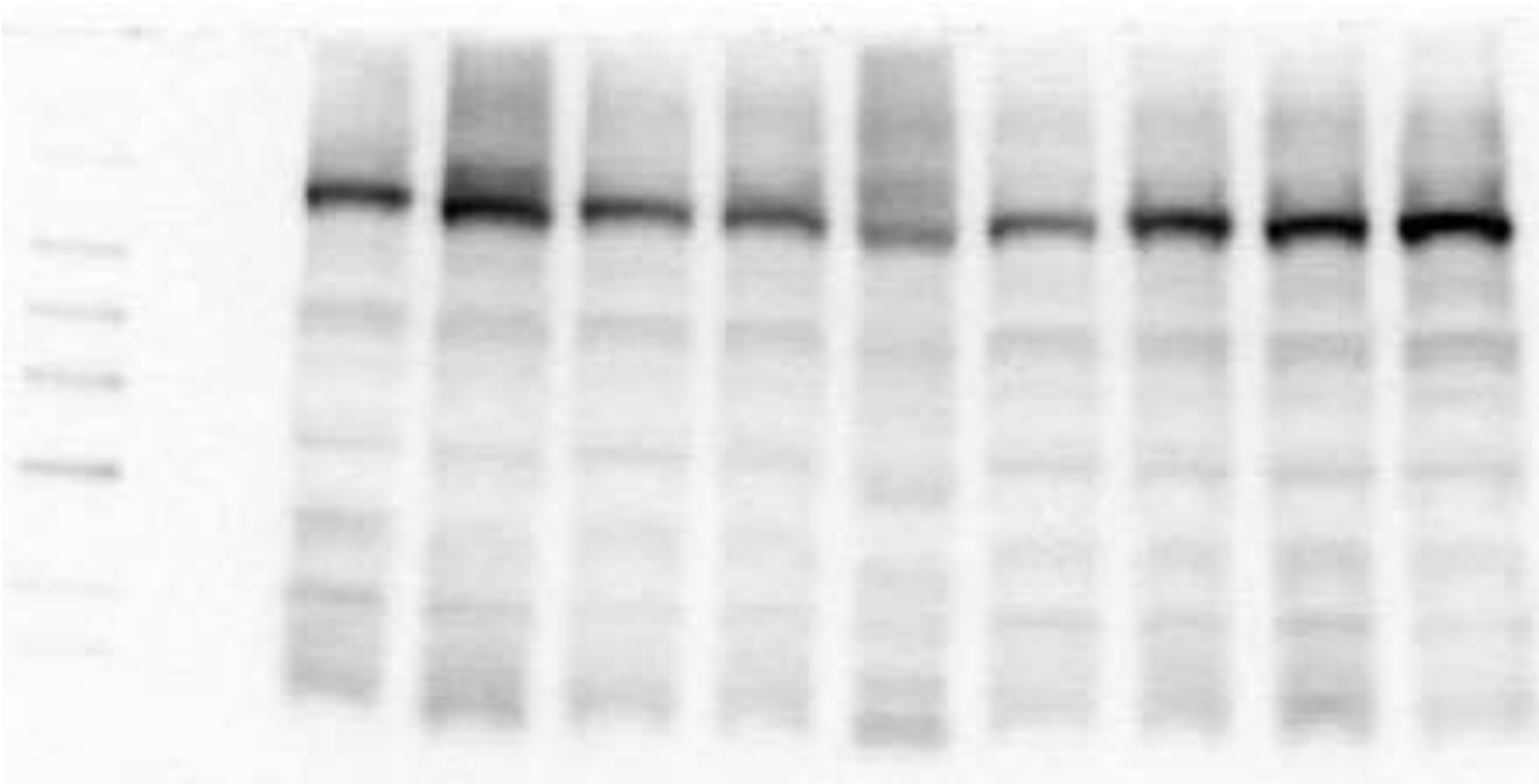

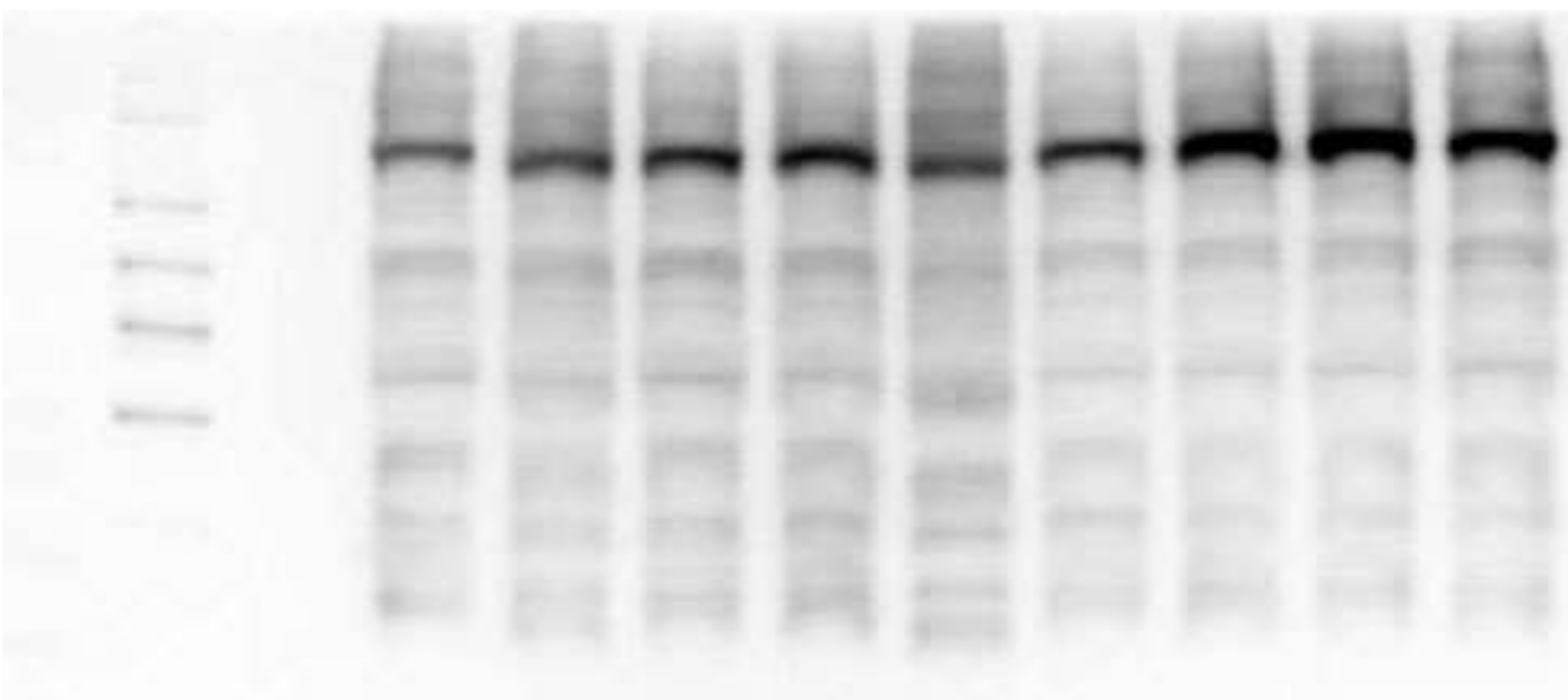

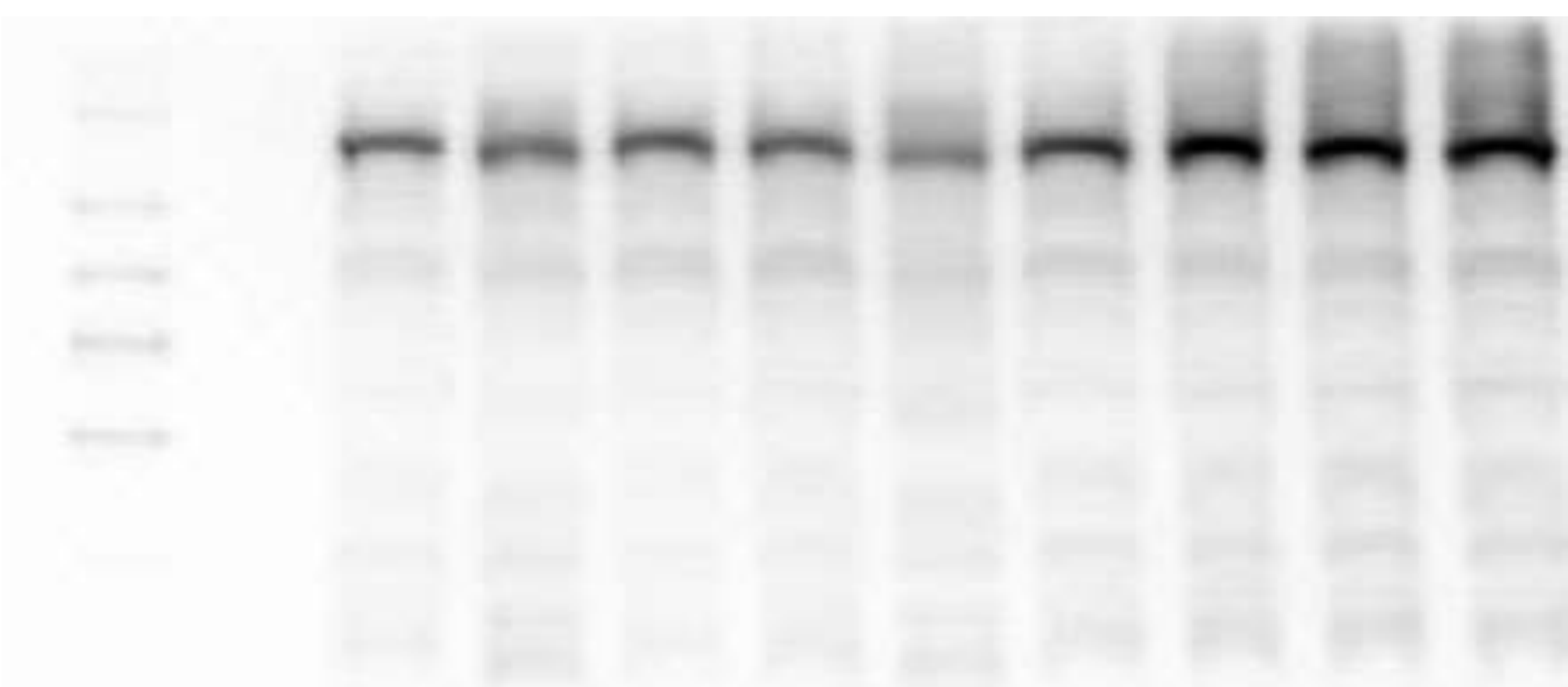

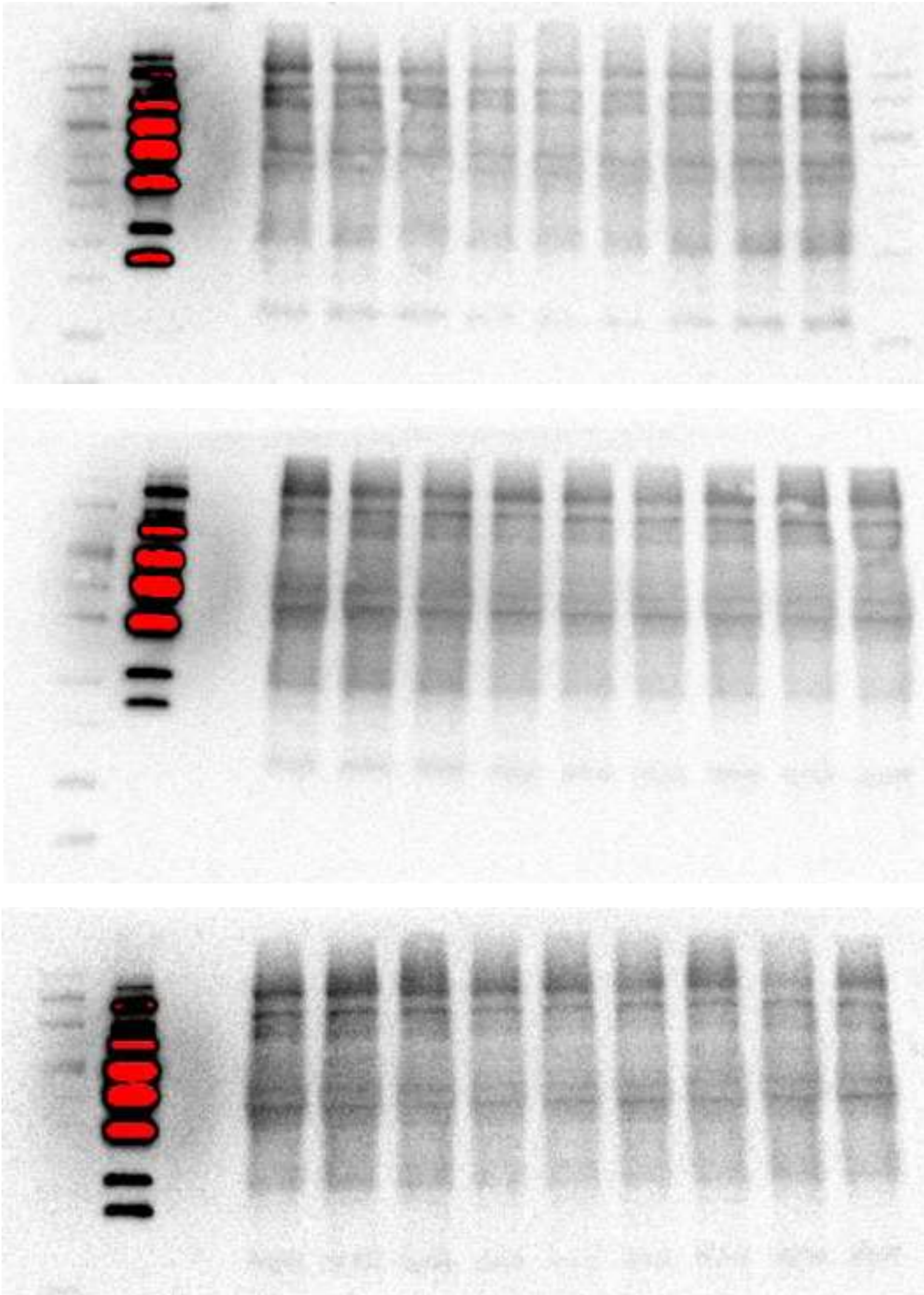

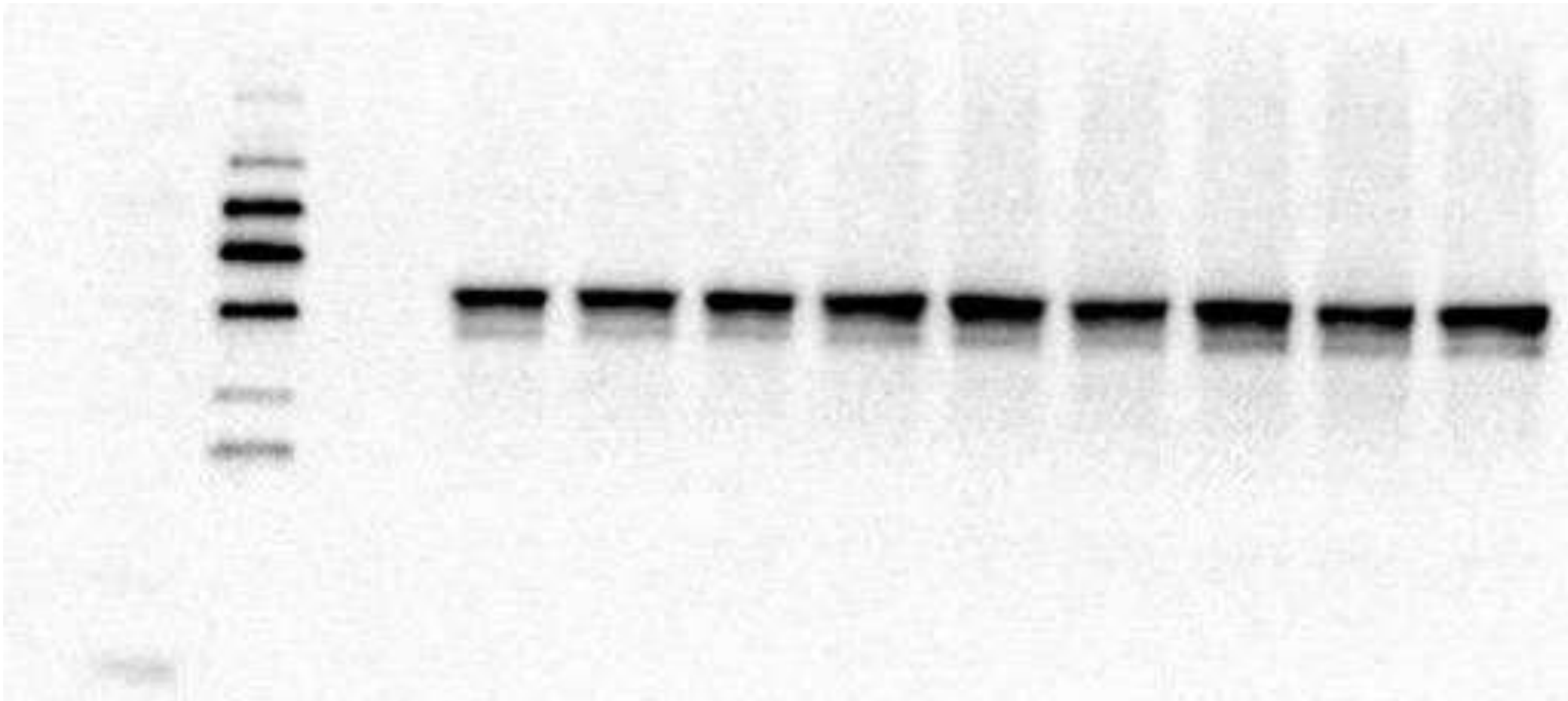

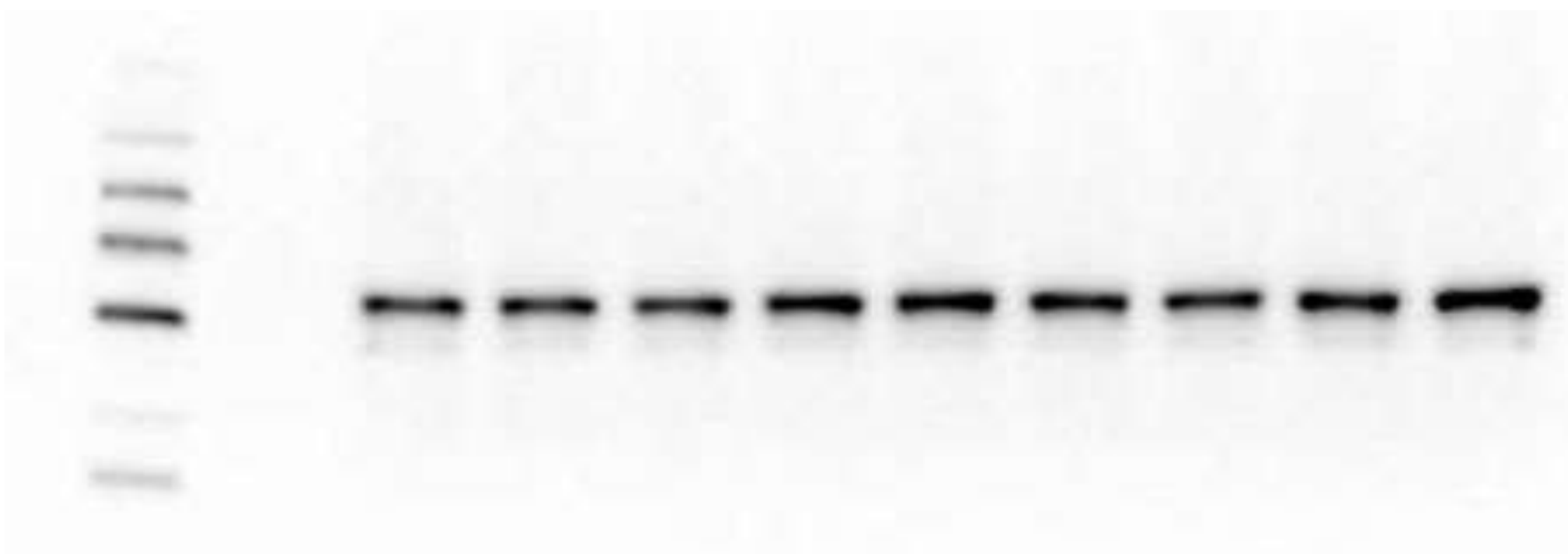

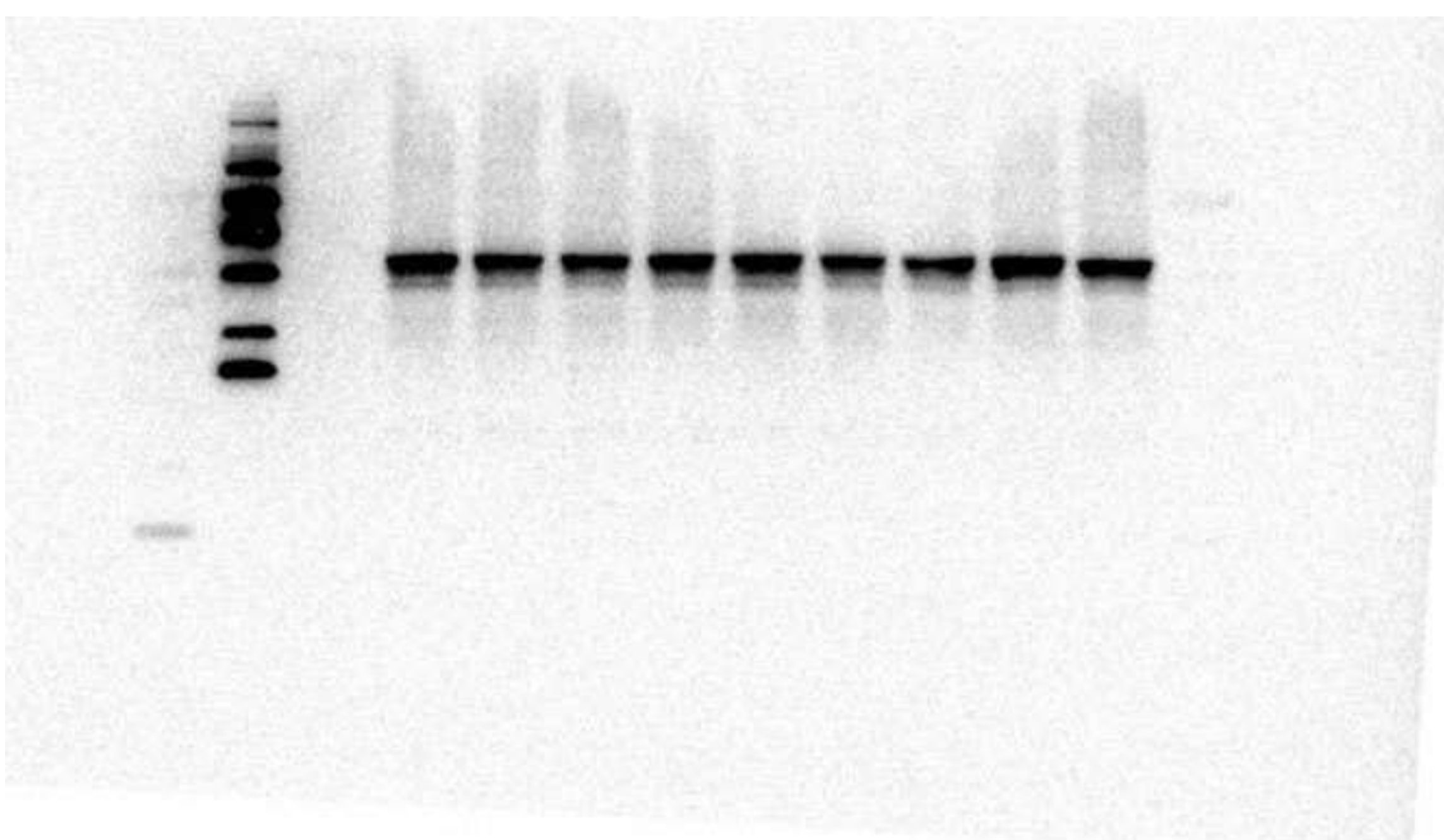

Supplement: Supplementary file 1 [file EMIDDT-26-E18715303402719_SD1.pdf]
